# Application Study of Comprehensive Forecasting Model Based on Entropy Weighting Method on Trend of PM_2.5_ Concentration in Guangzhou, China

**DOI:** 10.3390/ijerph120607085

**Published:** 2015-06-23

**Authors:** Dong-jun Liu, Li Li

**Affiliations:** Shenzhen Graduate School, Harbin Institute of Technology, Shenzhen 518055, China; E-Mail: laueastking3168@163.com

**Keywords:** PM_2.5_, comprehensive forecasting model, entropy weighting method, haze-fog

## Abstract

For the issue of haze-fog, PM_2.5_ is the main influence factor of haze-fog pollution in China. The trend of PM_2.5_ concentration was analyzed from a qualitative point of view based on mathematical models and simulation in this study. The comprehensive forecasting model (CFM) was developed based on the combination forecasting ideas. Autoregressive Integrated Moving Average Model (ARIMA), Artificial Neural Networks (ANNs) model and Exponential Smoothing Method (ESM) were used to predict the time series data of PM_2.5_ concentration. The results of the comprehensive forecasting model were obtained by combining the results of three methods based on the weights from the Entropy Weighting Method. The trend of PM_2.5_ concentration in Guangzhou China was quantitatively forecasted based on the comprehensive forecasting model. The results were compared with those of three single models, and PM_2.5_ concentration values in the next ten days were predicted. The comprehensive forecasting model balanced the deviation of each single prediction method, and had better applicability. It broadens a new prediction method for the air quality forecasting field.

## 1. Introduction

With the development of industry and the consumption of fossil fuels, air quality is worsening. In recent years, haze-fog pollution has occurred frequently in many parts of China [1]. The average concentrations of PM_2.5_, which is the main influence factor of haze-fog, in some areas of China are more than the average annual values in World Health Organization standard, which is 10 µg/m^3^ [2]. The haze-fog pollution goes from the local environmental factor to a nationwide environmental disaster. Especially in January 2013, there was extended haze-fog weather in the mid-eastern part of China. In the following months, the haze-fog pollution ranged from Beijing Tianjin Hebei region to the Yangtze River Delta region [3]. The spread of wide range of haze-fog caused a public panic, and caused serious impact on the normal production and operation. Air pollution is not only a threat to public health, which affects social stability, but also a bottleneck to economic development of many places [4]. The haze-fog pollution has negative effects on the environment, climate, human health, economic and other aspects, such as chronic diseases, respiratory and cardiac diseases, visibility reduction, damage of natural and agricultural systems and traffic accidents in the land, waterways, and air [5,6].

The most important factor for the formation of haze-fog pollution in the atmosphere is PM_2.5_. PM_2.5_ can suspend in the air for a long time. PM_2.5_ is particulate matter with an aerodynamic diameter ≤ 2.5 μm. It has been regulated in developed countries such as the USA, Australia, and some European countries [7,8]. In order to analyze the atmospheric environment pollution in China quantitatively and protect the living environment, new “Ambient Air Quality Standards (GB 3095-2012)” were introduced by the Ministry of Environmental Protection of China [9]. According to the new Ambient Air Quality Standards, Sulfur dioxide (SO_2_), Nitrogen dioxide (NO_2_), PM_10_, Ozone (O_3_), Carbon monoxide (CO) and PM_2.5_ were set as the six basic monitoring indicators, and released in real-time. “Air Quality Index” (AQI) was introduced to replace the earlier “Air Pollution Index” (API) at the same time. In the new ambient air quality standards, PM_2.5_ was added as a monitoring indicator and it is a key influencing factor.

Severe haze pollution and PM_2.5_ attracted widespread attention of scholars. Some researchers argued that the haze-fog formation was closely connected with the chemical reactions of pollutants in the planetary boundary layer and thermal and dynamic processes in the atmospheric environment [10,11]. Liu *et al*. (2013) and Zhang *et al*. (2013) also believed that the haze-fog formation might be influenced by primary pollutant emissions, anti-cyclone synoptic conditions, and the boundary layer height [12,13]. The major components of PM_2.5_ were nitrate, secondary sulfate, and organic aerosols in the haze-fog pollution in Shanghai, China [14]. The haze-fog pollution was extremely serious during the winter in central and eastern China, and the emission of coal combustion for heating and stagnant meteorological environment conditions affected the haze-fog greatly [15,16].

Because the atmosphere was seriously polluted, studies on prediction of concentration of important indicators in the atmosphere and analysis on air quality trends have important theoretical and practical significance. Soltani *et al*. (2007) developed the time-series model to forecast climatic fluctuations [17]. Autoregressive (AR) models, moving average (MA) models or autoregressive moving average (ARIMA) models were used in air-pollution modeling to predict and analyze the time series data [18,19]. However, in respect of the statistical analysis of air pollutant concentrations, the present works mainly focus on the future prediction and analysis of common indicators, such as NO_2_, O_3,_ and PM_10_. Chelani and Devotta (2006) used the ARIMA model to forecast the NO_2_ concentration in Delhi, India [20]. Prybutok and Mitchell (2000) developed the neural network model for forecasting daily maximum ozone levels [21]. Stadlober *et al*. (2008) presented the forecasting model to analyze the performance and quality of PM_10_ [22]. By contrast, the new indicator PM_2.5_, which was the main influencing factor of haze-fog pollution in China, has not been forecasted and analyzed.

In this study, PM_2.5_ was set as the research indicator, and the time series data of PM_2.5_ concentration were analyzed and forecasted. Three methods, that is, the ARIMA model, ANNs model, and Exponential smoothing method were used to forecast the time series data of PM_2.5_ concentration. Their results were combined with the entropy weighting method, and the comprehensive forecasting model was developed based on combination forecasting ideas. The comprehensive forecasting model was applied to predict and analyze the time series data of PM_2.5_ concentration in Guangzhou, China quantitatively. The trend of haze-fog pollution in Guangzhou was analyzed. The results were expected to provide a quantitative basis for the management and control of the haze pollution.

## 2. Related Theory

### 2.1. ARIMA Model

The Autoregressive Integrated Moving Average Model is an important time series prediction method. It was presented by Box and Jenkins in 1970s [23]. The basic ideas of the ARIMA model are as follows. In the ARIMA model, the time series data of the prediction object are regarded as a stochastic sequence, and this sequence is fitted with some mathematical models. Once this model is identified, the future values would be predicted by the time series of past and present values [24]. The ARIMA model can be divided into three types: (1) The autoregressive model (AR model), where *p* is the number of self-regression items; (2) The moving average model (MA model), where *q* is the number of moving average items; (3) The autoregressive integrated moving average model, that is, ARIMA (*p*, *d*, *q*), where *d* is the difference of frequency of time series data that become the stationary difference, and *d* is generally less than 2 in the practical application [25].

Assuming the random variable *Y_t_* was an observation value at the time *t* (*t* = 1, 2, ⋯, *n*). Then a series of *Y_t_* constitute a stochastic process. The ARIMA (*p*, *d*, *q*) model can be written as *Y_t_*~ARIMA (*p*, *d*, *q*), and its definition is as follows.

(1) $${\text{φ}}_{p}(B)W_{t}={\text{θ}}_{q}(B){\text{ε}}_{t}$$

where

φ*_p_*(*B*) = 1 − φ_1_*B* − φ_2_*B*^2^ − … − φ*_p_B^p^*

*W_t_* = (1 − *B*)*^d^Y_t_*

θ*_q_*(*B*) = 1 − θ_1_*B* − θ_2_*B*^2^ − … − θ*_q_B^q^*

ε*_t_* is white noise, and ε*_t_*~N(0, σ_a_^2^); *p*, *d* and *q* are non-negative integers; *B* is the moving operator, and *BY_t_* = *Y_t_*_-1_; φ_1_, φ_2_, …, φ*_p_* are the autoregressive parameters, while θ_1_, θ_2_, …, θ*q* are the moving average parameters.

The modeling processes of ARIMA model are as follows.

(1) Sample pretreatment. The establishment of the ARIMA model requests that the time series data should be stationary stochastic process. Thus the data should be tested for stationary before modeling.
(2) Pattern recognition. After the differential transform for the non-stationary time series, the key step is to determine the order of the ARIMA model. There are four methods to determine the order: (i) Auto Correlation Function (ACF) and Partial Auto Correlation Function (PACF) method; (ii) Final Prediction Error (FPE) method; (iii) Aikake Information Criterion (AIC) method; (iv) Aikake Information Corrected Criterion (AICC) method. The ACF and PACF method were used to master the direction of the general model to determine the order in this study.
(3) Model testing. After the order determination and parameter estimation, the applicability of the model established should be tested. If the model error is white noise, the obtained model is qualified. Otherwise, the order re-determination and parameter re-estimation are needed.
(4) Prediction. The time series data are forecasted in this step. The processes of model identification, parameter estimation, and model diagnosis are often improved gradually. The initial choices need to be constantly adjusted according to concrete problems.

The ARIMA model can find out the characteristics and trends of the variables from the time series data, and forecast the future values effectively. The ARIMA model is a prediction method with a good statistical theory, and has the advantages of high accuracy, and strong adaptive ability. It is used in many fields, and has wide applications [26,27].

### 2.2. Artificial Neural Networks Model

**Figure 1:**
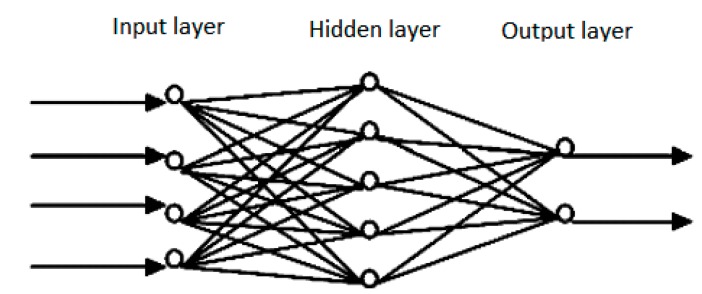
Structure of artificial neural network model.

The Artificial Neural Network model has been a hot research issue in the field of artificial intelligence since the 1980s. It can simulate the human brain neural networks for information processing, and construct different network models according to different connection ways. In recent years, research on the artificial neural networks developed, and great progress has been made. It is widely used in many fields, such as pattern recognition, intelligent robots, automatic control, biological, medical, economic etc. [28,29]. It has successfully solved many practical problems which are difficult to solve by modern computers, and shows a good intelligent characteristics [30]. The artificial neural network model is generally composed of the input layer, the hidden layer and the output layer, and its structure is shown as Figure 1.

The artificial neural network model has good characteristics of nonlinear combination, and is a global approximation network. It has strong learning ability, and can achieve nonlinear mapping between the input and output [31]. Artificial neurons in the ANNs model, as a simple processor, can sum the coming signal with appropriate weights, and its general expression is:

(2) $$y=\sum_{i=1}^{n}w_{i}x_{i}+b$$

where *x_i_* (*i* = 1, 2, …, *n*) are the input data; *w_i_* (*i* = 1, 2, …, *n*) are the weights; *b* is a threshold value, *y* is the output result.

The ANNs model can solve a lot of problems about the nonlinear system, such as function approximation, system identification. The choice of transfer functions and sample pretreatment should be paid more attention while modeling. The MATLAB neural network toolbox is very functional. It provides many functions of the design, training, and simulation of neural network model. The users can just call the functions according to their needs to design and simulate the neural network model facilitates, and this exempts the troubles of writing the complex and huge algorithms and programs. The MATLAB neural network toolbox was utilized to develop the ANNs model in this study.

### 2.3. Exponential Smoothing Method

The Exponential smoothing method is one of the important time series forecasting methods. It has a simple principle and good applicability. This method could not only be used for short-term prediction, but also had a better effect on the medium term or long term prediction problems. The basic prediction ideas are as follows. The average value of the first few periods is set as the initial value of the prediction period. Then when one novel observation value occurs, the earliest observation value would be removed from the initial few periods, and the novel observation value would be added. The novel prediction value can be obtained according to the novel observation value, the initial prediction value and weight of the latest observation value [32]. The Exponential smoothing method can eliminate the accidental changes of time series data, and enhance the importance of recent data as well.

The Brown quadratic polynomial exponential smoothing method was employed to predict the PM_2.5_ concentration time series data in this study. This method could track non-linear trend changes well. Its equation is:

(3) $$Y_{t+m}=a_{t}+b_{t}m+0.5c_{t}m^{2}$$

where *Y_t+m_* is the prediction value at the time *t* + *m* (*t* = 1, 2, …, *n*); *m* is the prediction step; *a_t_*, *b_t_* and *c_t_* are the parameters to be estimated, and they could be estimated according to the original time series data.

$$a_{t}=3S_{t}^{\prime }-3S_{t}^{\prime \text{​}\prime }+S_{t}^{\prime \text{​}\prime \text{​}\prime }$$

$$b_{t}=\text{α}\left[(6-5\text{α})S_{t}^{\prime }-(10-8\text{α})S_{t}^{\prime \text{​}\prime }+(4-3\text{α})S_{t}^{\prime \text{​}\prime \text{​}\prime }\right]/[2\cdot {\left(1-\text{α}\right)}^{2}]$$

$$c_{t}={\text{α}}^{2}(S_{t}^{\prime }-2S_{t}^{\prime \text{​}\prime }+S_{t}^{\prime \text{​}\prime \text{​}\prime })/{\left(1-\text{α}\right)}^{2}$$

where $S_{t}^{\prime }=\text{α}x_{t}+(1-\text{α})S_{t-1}^{\prime }$, $S_{t}^{\prime \text{​}\prime }=\text{α}S_{t}^{\prime }+(1-\text{α})S_{t-1}^{\prime \text{​}\prime }$, $S_{t}^{\prime \text{​}\prime \text{​}\prime }=\text{α}S_{t}^{\prime \text{​}\prime }+(1-\text{α})S_{t-1}^{\prime \text{​}\prime \text{​}\prime }$; *x_t_* are the original time series data; α is the weight of the latest observation value, and it could take the experience value α=0.15 [32].

### 2.4. Entropy Weighting Method

In information science theory, entropy is a very important concept. Information entropy is a measure of the degree of disorder of system information, and can measure the amount of useful information of the data [33]. The basic idea of the entropy weighting method is as follows. When the data of one object show great differences, according to information theory, its entropy would be low. This shows the object could contribute much useful information, so its weight should be set high; otherwise, the weight should be set low correspondingly [34]. Entropy weighting method is an objective weighting method. In this study, the entropy weighting method was used to weight the results of three prediction methods. The processes of determining weights are as follows: 

(i) The original data of all objects should be normalized to eliminate effects of dimension. For the benefit object, the higher its value, the greater its impact. Its equation is:

(4) $$r_{ij}=\frac{x_{ij}-\underset{i}{\min}\{x_{ij}\}}{\underset{i}{\max}\{x_{ij}\}-\underset{i}{\min}\{x_{ij}\}}$$

For the cost object, the lower its value, the greater its impact. Its equation is:

(5) $$r_{ij}=\frac{\underset{i}{\max}\{x_{ij}\}-x_{ij}}{\underset{i}{\max}\{x_{ij}\}-\underset{i}{\min}\{x_{ij}\}}$$

where, *x_ij_* (*i* = 1, 2, …, *m*, and *j* =1, 2, …, *n*) is the observation value of the *j*-th object on the *i*-th object, and *r_ij_* is the dimensionless value that has been normalized.

(ii) The entropy *p_i_* of the *i*-th object could be defined as:

(6) $$p_{i}=-k\;\sum_{j=1}^{n}f_{ij}\ln f_{ij}$$

where $f_{ij}=r_{ij}/\sum_{j=1}^{n}r_{ij}$, *k* = 1/ln *n*, *i* = 1, 2, …, *m*. While *f_ij_* = 0, we set *f_ij_* ln *f_ij_* = 0.

(iii) The weight of the *i*-th object λ*_i_* could be defined according to the entropy theory:

(7) $${\text{λ}}_{i}=\frac{1-p_{i}}{m-\sum_{i=1}^{m}p_{i}}$$

where 0 ≤ λ*_i_* ≤ 1, and $\sum_{i=1}^{m}{\text{λ}}_{i}=1$.

## 3. Simulation Data and Qualitative Trend Analysis

The comprehensive forecasting model was utilized to predict PM_2.5_ concentration in the atmosphere in Guangzhou city in China. Guangzhou city is the capital of Guangdong Province in China, and is the center of political, economic, science and technology, education and culture of Guangdong Province. Guangzhou in located in the south of Guangdong Province in southern China and at the northern margin of the Pearl River Delta. Guangzhou is on the verge of the South China Sea, with significant characteristics of an oceanic climate. With the Tropic of Cancer crossing through the north of the city, it is warm and rainy, with plenty of heat, small temperature difference and a long summer and other climatic characteristics. Guangzhou has the characteristics of typical southern coastal cities in China, and studies in this manuscript had important significance for the studies on the haze-fog pollution in this category of cities. The original data were the time series data from 2 December 2013 to 21 January 2015 in Guangzhou city [35]. They were from the China National Environmental Monitoring Center. They were the values of 24-h averages.

The factors that influenced the changes of PM_2.5_ concentrations included two aspects: one was the basis concentration that was determined by the actual air quality, the other was the impact on PM_2.5_ concentration from the external meteorological environment and random factors. With the changes of sunshine, temperature, and pressure the concentration of PM_2.5_ would change along with the time. The external environment change, such as increasing automobile exhaust quantity and more garbage incineration would also affect the concentration of PM_2.5_.

**Figure 2:**
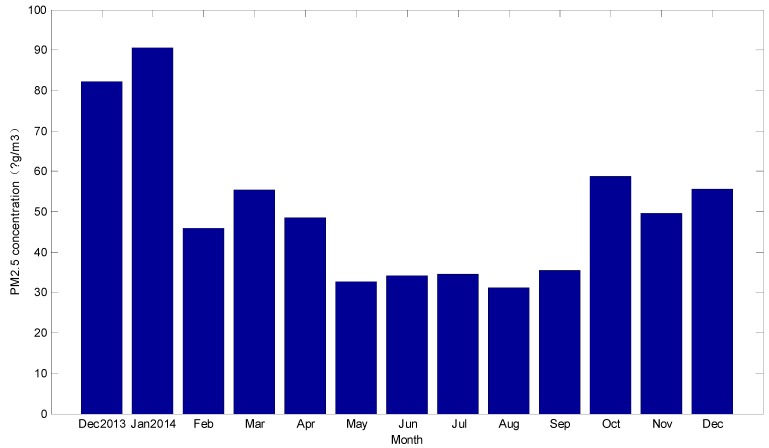
Average of PM_2.5_ concentrations per month.

The long-term trend of the concentration of PM_2.5_ over one year was investigated. It was the trend that was affected by some fundamental factors for a long period. The averages of every month of the PM_2.5_ concentration were calculated, as shown in Figure 2.

According to Figure 2, averages of PM_2.5_ concentrations in the winter of 2013 and 2014 were higher than those in the summer of 2014. In addition, the averages of PM_2.5_ concentration in the winter of 2013 were higher than those of 2014. A seasonal change rule of PM_2.5_ refers to PM_2.5_ concentrations showing regular changes in one year along with the change of season. The seasonal variations were significant according to Figure 2, and the averages were the highest in winter, the lowest in summer. This may mainly be affected by the seasonal temperature, precipitation, and other meteorological factors. The summer precipitation is substantial, and rainwater can bring some of the particulate matter to the ground. In addition, the weather is warm in the summer, and the people in China would not burn coal to keep warm. Thus PM_2.5_ concentrations were relatively low. In addition, summer weather conditions such as: a high atmospheric boundary layer, frequent precipitation, *etc*. were conducive to clear the particles. However, temperatures in winter were always low in China, and the atmospheric pressure was high. The concentrations of PM_2.5_ in winter were generally high. Meanwhile wind speed, relative humidity, and other meteorological factors would also affect the concentration of PM_2.5_.

## 4. Simulation Results Based on Comprehensive Forecasting Model

### 4.1. Comprehensive Forecasting Model

For the issue of time series data prediction, there are a variety of forecasting models and methods, such as regression analysis, the ARIMA model, gray forecasting system, ANNs and so on. While their modeling mechanism and application conditions are different, they all have some limitations for a certain prediction problem in the application fields. In 1969, Bates and Granger proposed an idea of “combination forecasting” on “Operations Research Quarterly” for the first time [36]. It began a systematic study on “combination forecasting” issue. Several forecasting methods were combined into one comprehensive prediction model. In this way, a comprehensive description of the objective system could be made, and the combination forecasting model was used widely.

Different forecasting values could be obtained based on different prediction methods. We developed mathematical models based on the ARIMA model, the ANNs model and the Exponential smoothing method respectively, and combined the predictive values at the same time with the weights from the entropy weighting method. Thus the combination forecasting values could be obtained. The combination equation was:

(8) $${\hat{x}}^{\left(0\right)}=k_{1}\cdot {\hat{x}}_{1}^{\left(0\right)}+k_{2}\cdot {\hat{x}}_{2}^{\left(0\right)}+\cdots +k_{n}\cdot {\hat{x}}_{n}^{\left(0\right)}$$

where *k_1_* *+ k_2_* *+···+ k_n_* *=* 1, and *k_i_* ≥ 0 (*i* = 1, 2, ···, *n*) were the weights of each prediction sequence.

### 4.2. Simulation Results

Based on the algorithm of comprehensive forecasting model in Section 2.1, we programmed the MATLAB software platform according to the time series data of PM_2.5_ concentration. The ARIMA model was developed as follow:

φ(*p*) *W* (*t*) = θ(*q*) ε(*t*) (9)

where

φ(*p*) = 1 − 0.08989 *p*^−1^ − 0.7232 *p*^−2^

θ(*q*) = 1 + 0.6083 *q*^−1^ − 0.3337 *q*^−2^ − 0.2434 *q*^−3^

**Figure 3:**
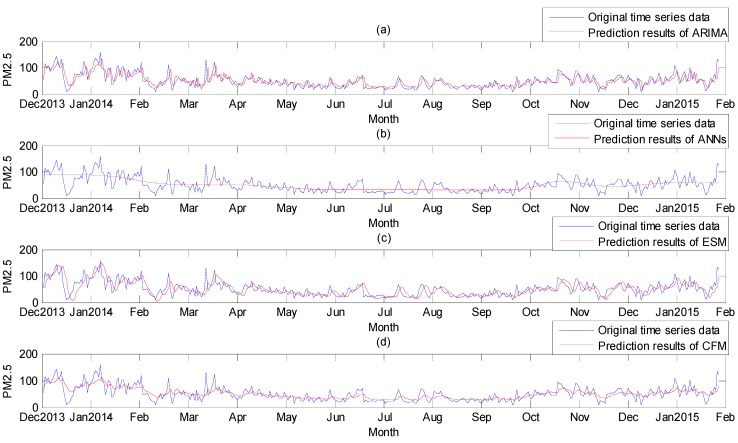
Comparison of original data and prediction results. (**a**) ARIMA model; (**b**) ANNs model; (**c**) ESM model; (**d**) Comprehensive forecasting model.

The model we developed was ARMA (2, 3) as the Equation (9). The prediction results were shown in Figure 3a.

Using MATLAB toolbox, the ANNs model was constructed. The input and output models were respectively “tansig” (Hyperbolic tangent sigmoid transfer function) and “purelin” (Linear transfer function) function. Number of neurons in the hidden layer was selected according to the principle that the sum of squares of prediction errors was the smallest, and the number of neurons in the hidden layer was selected as 5 finally. The training step was set as 20,000, and the error precision was 0.001. Then the prediction values were obtained. They were shown in the Figure 3b.

According to formulas in Section 2.3, the Exponential Smoothing Model was established based on the time series data and the results were shown in Figure 3c.

From the entropy weighting method, the weights of the three methods were respectively: *k*_1_ = 0.2399, *k*_2_ = 0.5419 and *k*_3_ = 0.2182. The results of the three methods were combined with the weights. The results of the comprehensive forecasting model were obtained, as were shown in Figure 3d.

From the four figures we could see, the prediction results of each method were different, and they all had their own characteristics. The results of the ARIMA model and the ESM model tracked the original time series data, but their results might lag behind the original data. The trend of results of ANNs followed the original time series data, and its results were near the means of the data sequence. The prediction results of the comprehensive forecasting model were the combination of results of three methods. The original data of PM_2.5_ concentration were severely affected by the external meteorological environment and random factors. The original data were with great fluctuation, and the fluctuation was often not regular. The ANNs model excluded these irregular changes and seized the basic trends of the time series data. We could believe that the results of CFM model followed the trend of the original data along with the results of ANN model, and meanwhile they fluctuated around the means of the original data sequence according to the results of the ARIMA and ESM models. Thus the prediction results of the comprehensive forecasting model also tracked the original time series data, and its curve fluctuated with the curve of original data.

### 4.3. Accuracy Test

In order to investigate the accuracy and precision of the prediction results, the results should be tested by error testing indexes. The error testing indexes included Mean Absolute Error (MAE), Mean Percentage Error (MPE), Root Mean Square Error (RMSE), Theil inequality coefficient, bias ratio and variance ratio. The calculation formulas and functions of the error testing indexes were shown in Table 1. The various error testing indexes were calculated, and the results were shown in Table 2.

**Table 1:** Calculation formulas of error testing indexes.

No.	Index	Formula	Function
1	MAE	$MAE=\frac{1}{n}\sum_{i=1}^{n}\left|y_{i}-{\hat{y}}_{i}\right|$	It can describe the system errors, and is an absolute index.
2	MPE	$MPE=\frac{1}{n}\sum_{i=1}^{n}\left|\frac{y_{i}-{\hat{y}}_{i}}{y_{i}}\right|$	It can describe the system errors, and is a relative index and dimensionless.
3	RMSE	$RMSE=\sqrt{\frac{1}{n}\sum_{i=1}^{n}{\left(y_{i}-{\hat{y}}_{i}\right)}^{2}}$	It can describe the system errors, and is an absolute index.
4	Theil inequality coefficient	$U=\frac{\sqrt{\frac{1}{n}\sum_{i=1}^{n}{\left(\frac{y_{i}-{\hat{y}}_{i}}{y_{i}}\right)}^{2}}}{\sqrt{\frac{1}{n}\sum_{i=1}^{n}{\hat{y}}_{i}^{2}}+\sqrt{\frac{1}{n}\sum_{i=1}^{n}y_{i}^{2}}}$	It can describe the system errors, and is a relative index and dimensionless.
5	Bias ratio	$BR=\frac{{\left(\bar{y}-\bar{\hat{y}}\right)}^{2}}{\frac{1}{n}\sum_{i=1}^{n}{\left(y_{i}-{\hat{y}}_{i}\right)}^{2}}$	It can measure the deviation degree of the average between the forecasting sequence and original sequence.
6	Variance ratio	$VR=\frac{{\left(s_{y}-s_{\hat{y}}\right)}^{2}}{\frac{1}{n}\sum_{i=1}^{n}{\left(y_{i}-{\hat{y}}_{i}\right)}^{2}}$	It can measure the deviation degree of the variance between the forecasting sequence and original sequence.

Note: In Table 1, $y_{i}$ (*i* = 1, 2, ⋯ *n*) were the actual observation values; ${\hat{y}}_{i}$ were the prediction values; $\bar{y}$ and $\bar{\hat{y}}$ were the averages of $y_{i}$ and ${\hat{y}}_{i}$; $s_{y}$ and $s_{\hat{y}}$ were the standard deviation of $y_{i}$ and ${\hat{y}}_{i}$.

**Table 2:** Error testing index.

No.	Index	ARIMA	ANNs	ESM	CFM
1	MAE (µg/m^3^)	12.6578	15.4849	15.8016	13.3090
2	MPE	0.3212	0.4159	0.3821	0.3522
3	RMSE (µg/m^3^)	17.4596	20.7186	21.3586	18.0247
4	Theil inequality coefficient	0.0050	0.0071	0.0058	0.0060
5	Bias ratio	6.12 × 10^−^^7^	2.69 × 10^−^^5^	2.56 × 10^−^^4^	5.70 × 10^−^^5^
6	Variance ratio	0.1212	0.2201	0.0021	0.2338

The MAE, MPE, RMSE and Theil inequality coefficient can describe the system errors, and indicate the dispersion of prediction results and original sequence. These four indexes are as small as possible for good prediction results. In Table 2, from these four indexes, results of the ARIMA model were the best, and the CFM was slightly lower than those of ARIMA model. Results of the ANNs and ESM were worse than those of the former two models. The bias ratio and variance ratio measure the deviation degree of the average and variance between the forecasting sequence and the original sequence. These two indexes were also as small as possible for good forecasting results. The bias ratio of the ARIMA model was the best, while that of the ESM was the worst; the variance ratio of the ESM was the best, while that of the CFM was the worst.

To sum up, the accuracy of the ARIMA model was the best for the historical data in this study, while the accuracy of the CFM was close to it. They were significantly higher than that of other two methods. This showed that, for a particular sequence, the applicability of one prediction method might just suit to it, and its forecasting accuracy might be better than other methods. However, the applicability of the single prediction method was often limited, and not universal. We combined multiple methods and developed a combination forecasting model. The combination forecasting model can balance the deviation of each single prediction method, and had better applicability. High accuracy could also be achieved at the same time. Thus the comprehensive effect of the combination forecasting model was good in the practical applications.

### 4.4. Prediction of Next Ten Days

The comprehensive forecasting model was applied to predict PM_2.5_ concentrations in the next ten days in Guangzhou, compared with the ARIMA model, ANNs model and ESM model. Their results were shown in Table 3. The actual observation values were obtained from the website “Historical data of PM_2.5_” on the internet [35], where PM_2.5_ concentrations were updated in real-time. The important error testing indexes were calculated to evaluate the prediction accuracy, as were shown in Table 4.

**Table 3:** Prediction results of next ten days.

No.	Date	Actual Observation Value (µg/m^3^)	ARIMA (µg/m^3^)	ANNs (µg/m^3^)	ESM (µg/m^3^)	CFM (µg/m^3^)
1	2015/1/22	58.2	101.6861	61.6869	114.865	82.8862
2	2015/1/23	64.4	37.9755	61.6923	98.6254	64.0614
3	2015/1/24	73.6	56.3128	61.6964	89.1382	66.3927
4	2015/1/25	68.8	43.7694	61.6993	86.038	62.7086
5	2015/1/26	68.3	41.9564	61.7012	81.2968	61.2402
6	2015/1/27	64.8	43.2804	61.7021	77.4207	60.7125
7	2015/1/28	49.3	38.7611	61.7022	72.8985	58.6417
8	2015/1/29	51.7	41.4545	61.7016	62.6025	57.0409
9	2015/1/30	32.8	38.5742	61.7004	56.4024	54.9964
10	2015/1/31	35.6	40.069	61.6986	43.6473	52.5709

**Table 4:** Error testing indexes of prediction results of next ten days.

No.	Index	ARIMA	ANNs	ESM	CFM
1	MAE(µg/m^3^)	19.1119	11.2298	21.5435	10.3321
2	MPE	0.3188	0.2571	0.3987	0.2229
3	RMSE(µg/m^3^)	22.2286	14.2652	25.5865	12.8903
4	Theil inequality coefficient	0.0033	0.0032	0.0034	0.0025
5	Bias ratio	0.1417	0.1203	0.7089	0.1739
6	Variance ratio	0.0522	0.8789	0.0616	0.1757

From Table 4 we could see MAE, MPE, RMSE and Theil inequality coefficient of the CFM were significantly less than those of other three methods in the numerical values. This showed that the combination forecasting values of CFM model were closer to the actual observation values. The prediction accuracy of the CFM model was higher than that of these three methods, and the results of the CFM model were more effective and reliable. However, the comprehensive forecasting model had some shortcomings. The workload of the comprehensive forecasting method might be heavier than that of the single prediction method.

## 5. Conclusions

Haze-fog was the most serious air pollution in 2013. The most important factor of haze-fog pollution was PM_2.5_. The sources of PM_2.5_ were wide, and its formation was complex. In order to reflect the trend of haze-fog pollution, it is very important to strengthen pollution prevention and control. The comprehensive forecasting model was developed based on three prediction models in this study. The time series data of PM_2.5_ concentration were forecasted by the ARIMA model, ANNs model, and ESM model. Their results were combined with weights from the entropy weighting method. Thus the combination forecasting results were obtained. The comprehensive forecasting model was applied to predict PM_2.5_ concentration in Guangzhou China, and good forecasting results were obtained. The results were with high accuracy compared with those of the three single methods. The combination forecasting model could make a balance of deviation of each single forecasting method, and overcome the applicability limitations of each single method. It broadened a new prediction method for the air quality forecasting field. This study could provide a scientific basis for the prevention and prediction of haze-fog pollution in the city, and provide a methodological basis for this kind of scientific research.
